# Transcriptomic Analysis of Myocardial Ischemia Using the Blood of Rat

**DOI:** 10.1371/journal.pone.0141915

**Published:** 2015-11-05

**Authors:** Jincai Hou, Jianhua Fu, Dan Li, Xiao Han, Lei Li, Wenting Song, Mingjiang Yao, Jianxun Liu

**Affiliations:** 1 Institute of Basic Medical Sciences of Xiyuan Hospital, China Academy of Chinese Medical Sciences, Beijing, China; 2 Beijing Key Laboratory of Pharmacology of Chinese Materia Medica, Beijing, China; Harbin Medical University, CHINA

## Abstract

Myocardial ischemia is a pathological state of heart with reduced blood flow to heart and abnormal myocardial energy metabolism. This disease occurs commonly in middle aged and elderly people. Several studies have indicated that the rat was an appropriate animal model used to study myocardial ischemia. In this study, in order to gain insights into the pathogenesis of myocardial ischemia, we sequenced the transcriptomes of three normal rats as control and the same number of myocardial ischemia rats. We sequenced the genomes of 6 rats, including 3 cases (myocardial ischemia) and 3 controls using Illumina HiSeq 2000. Then we calculated the gene expression values and identified differentially expressed genes based on reads per kilobase transcriptome per million (RPKM). Meanwhile we performed a GO enrichment analysis and predicted novel transcripts. In our study, we found that 707 genes were up-regulated and 21 genes were down-regulated in myocardial ischemia rats by at least 2-fold compared with controls. By the distribution of reads and the annotation of reference genes, we found 1,703 and 1,552 novel transcripts in cases and controls, respectively. At the same time, we refined the structure of 9,587 genes in controls and 10,301 in cases. According to the results of GO term and pathway analysis of differentially expressed genes, we found that the immune response, stimulus response, response to stress and some diseases may be associated with myocardial ischemia. Since many diseases, especially immune diseases, are associated with myocardial ischemia, we should pay more attention to the complications which might result from myocardial ischemia.

## Introduction

Myocardial ischemia is a disease characterized by reduction of blood to the myocardial tissue, which has a high mortality and morbidity [1] and occurs frequently in elderly population. In the past, there have been many researches which focused on the molecular mechanisms of myocardial ischemia using different animal models. For instance, Ferrari R et al. used rabbits to study the metabolic derangement in ischemia heart disease[2]; Michael LH et al. built a murine model of myocardial ischemia and reperfusion[3]. Among the animal models, the rat model is the most commonly used animal model of myocardial infarction because the progression to heart failure in these rats is similar to what happens when a patient sustains a large myocardial infarction[4]. Recently, the number of studies related with myocardial infarction using rat models has been increasing. For instance, some researches explored the effect of a certain drug on myocardial infarction[5, 6], some investigated the effect of therapeutic methods against myocardial infarction[7], others focused on the potential role of a gene or protein in a rat myocardial infarction model[8].

Although there have been many studies about the mechanism of myocardial ischemia, the pathogenesis is still not very clear because most of these studies focused on the genes associated with specific pathways or conditions, such as apoptosis[9] and myocardial stress[10]. Along with the technology advancement, the cDNA expression arrays were used to identify the novel pathways associated with ischemia[11]. Despite of some information learnt from these studies using microarray technologies, the limits of microarray still exist, such as the extent of detection[12] and identification of novel genes. However, the appearance of RNA-seq[13] has greatly improved this situation. Compared with microarray, RNA-seq showed high reproducibility[14] and could not only quantify the expression of known genes, but also identify novel genes or transcripts. Recently, there have been many studies using RNA-seq which reveal new characteristics of transcriptomes in mice with heart failure[15, 16].

In our study, we performed a comprehensive transcriptome analysis using three normal rats and three myocardial ischemia rats. The results suggested that there were significant differences in expression levels of genes between normal rats and myocardial ischemia rats. Our study has deepened the understanding of the influence of transcriptional regulation on myocardial ischemia and enhanced our understanding of the pathogenesis of myocardial ischemia.

## Methods

### Animals and experimental design

The animals in our experiment were 6 male Wistar rats. The weights of them ranged from 220 to 240 g. The present study was carried out in strict accordance with the recommendations in the Guide for the Care and Use of Laboratory Animals of the National Institutes of Health. The protocol was approved by the Institute’s Animal Care and Use Committee of Experimental Animal Research Institute, Chinese Academy of Medical Sciences [Certificate Number: SCXK (Jing) 2009–0007] where there were veterinarians and scientists involved who are qualified in the evaluation of animal ethical issues. All surgery was performed with general anesthesia by using hydrate anesthesia, and all efforts were made to minimize suffering.

We divided these rats into two groups: one was for surgical ligation ischemia, the other was for sham group. The rats of surgical group were anaesthetized with 0.35ml/100g chloral hydrate. Then we opened the pericardium of rats and ligated the root of the left anterior descending coronary artery threading. After 40 minutes of ligation, we cut the left ventricular ischemic area of rats quickly and then froze them in liquid nitrogen to be detected. The rats of sham group were not ligated. The condition of the experimental rats was monitored every ten minutes after surgical ligation ischemia. There were not any unintended deaths as a result of the surgical procedures. At the end of the treatment rats were sacrificed by cervical dislocation under general anesthesia induced by hydrate anesthesia.

### RNA sequencing of samples

In this study, RNAs from three healthy control rats and three diseased treated samples were enriched by using the oligo (dT) magnetic beads. By using the fragmentation buffer, the mRNAs were fragmented into short fragments (about 200~700 bp), then the first-strand cDNAs were synthesized by random hexamer-primer using the mRNA fragments as templates. The double strand cDNAs were purified with QiaQuick PCR extraction kit. The library products were ready for sequencing analysis via Illumina Hiseq^™^ 2000. The sequence results were saved as FASTQ[17]. The sequence datasets have been uploaded to Sequence Read Archive (SRA) database and the accession numbers are SRR2491682 and SRR2505874.

### Quality analysis of sequence data

Before mapping the reads to genome, quality control was required. If the base composition percentage of a sample was balanced, resequencing was not required. In both control and case samples, the base composition percentages of them were balanced. Then, we filtered out the reads with adapters and with more than 10% unknown bases. In addition, the low quality reads (the percentage of low quality bases was over 50% in a read, we defined the threshold of quality was 10) were also discarded. After the steps below, the remaining reads were called “clean reads” and used for further analysis.

### Mapping reads to reference genome

In order to assess the effectiveness of selected mapping tool, we compared the SOAP2 and bowtie[18] and found that SOAP2 performed better at the total mapping reads percent and the unique mapping percent using same alignment criteria. Therefore, in this study, we mapped the reads to genome by SOAP2 with more than 5 mismatches. The expression of genes were calculated by Reads Per Kilobase transcriptome per Million reads mapped (RPKM)[19]. Then a method named ‘The significance of digital gene expression profiles’[20] was used to identify differentially expressed genes. We used FDR method to adjust the P-value corresponding to differential gene expression. If the FDR ≦0.001[21] and the absolute value log_2_ *Ratio*≧1, the gene was a significantly differentially expressed gene.

### Functional analysis of differentially expressed genes

The differentially expressed genes were annotated to GO terms in the Gene Ontology database (http://www.geneontology.org/). We utilized the cumulative hypergeometric algorithm to perform GO analysis. The method was described as follows:

$$P=1-\sum_{i=0}^{m-1}\frac{C_{M}^{i}C_{N-M}^{n-i}}{C_{N}^{n}}$$

(N was the number of all genes with GO annotation; n was the number of DEGs in N; M was the total number of genes contained in a certain GO terms; m was the number of DEGs in M). A GO term was defined as a significantly enriched GO term if the p-value corrected by Bonferroni Correction was smaller than 0.05. The process of pathway enrichment analysis of DEGs was similar to GO enrichment analysis.

### Predicting novel transcripts

We assembled transcripts with reads by cufflink[22]. By comparing our assembled transcripts with annotated genomic transcripts from reference genome, we tried to find novel transcripts. In our study, if a transcript met three following requirements, it could be reported as a novel transcript: i) the transcript must be at least 200bp away from annotated gene; ii) the length of the transcript was over 180bp; iii) the sequencing depth was no less than 2.

## Results

### Transcriptome sequencing of samples

Six rats in our study were provided by Beijing Hua Fukang Biological Technology Limited Company. All of these rats were male. Paired-end cDNA libraries for each RNA sample were sequenced by Illumina Hiseq^™^ 2000.

### Alignment of reads

We used SOAP2[23] to map the clean reads to reference sequences with no more than 5 mismatches. In our study, the reference sequences could be one of the entire genome, high reliability transcripts(e.g., RefSeq, CCDS or VEGA transcripts) and lower reliability transcripts sources(e.g., GenBank transcripts or ESTs). Therefore our alignment results would be presented for genome and gene, respectively. The statistics were showed in Tables 1 and 2 below. The number of reads for each sample was 27,488,888. For genome, 76.33% and 77.53% of total reads were mapped to the reference genome in case and control samples respectively. For genes, 42.13% and 42.52% of total reads were mapped to the reference genes in case and control samples respectively. We also provided the distribution of sequence quality scores in the S1 and S2 Figs for both cases and controls.

**Table 1:** Statistics of reads mapped to reference genome.

	Case	Control
Total reads	27,488,888(100%)	27,488,888(100%)
Mapped reads	20,981,954(76.33%)	21,313,098(77.53%)
Unique match reads	17,796,433(64.74%)	18,279,163(66.50%)
Unmapped reads	6,506,934(23.67%)	6,175,790(22.47%)

**Table 2:** Statics of reads mapped to reference genes.

	Case	Control
Total reads	27,488,888(100%)	27,488,888(100%)
Mapped reads	11,580,711(42.13%)	11,689,116(42.52%)
Unique match reads	11,042,257(40.17%)	11,194,176(40.72%)
Unmapped reads	15,908,177(57.87%)	15,799,772(57.48%)

### Differentially expressed genes

We applied a strict algorithm referring to ‘The significance of digital gene expression profile’ to identify differentially expressed genes (details see Materials and Methods). In our study, genes with the FDR ≦0.001 and the absolute value log_2_ *Ratio*≧1 were treated as the significantly differentially expressed genes.

There were 15,116 genes expressed in case samples and 14,884 genes in control samples of which 639 genes were only expressed in control samples whereas 872 genes were only detected in case samples. According to our significant threshold, we got 728 significantly differentially expressed genes of which 707 genes were up-regulated and only 21 genes were down-regulated (the statistics of these genes were showed in Table 3). The top 10 up-regulated and down-regulated genes in myocardial ischemia rats were presented in Table 4 and the whole list of 728 differentially expressed genes can be found in S1 Table.

**Table 3:** The statics of genes expression.

	The number of genes	
	Case	Control
Total genes	15,116	14,884
Genes only expressed in case	872	-
Genes only expressed in control	-	639
DEGs which were up-regulated	707	-
DEGs which were down-regulated	21	-

**Table 4:** Top ten up- regulated and down-regulated genes in myocardial ischemia.

Ensembl gene ID	Gene symbol	Description	Chr	Start	End	Log2 Ratio(Case FPKM/Control FPKM)	Up or Down
ENSROG00000002792	Cxcl2	Chemokine(C-X-C motif) ligand 2	14	18,640,312	18,642,357	12.3797	Up
ENSRNOG00000038831	_	_	_	_	_	12.1845	Up
ENSRNOG00000025980	Gm14137	Predicted gene 14137	3	117,533,191	117,533,736	11.8366	Up
ENSRNOG00000024399	Cd300a	CD300A antigen	10	103,094,716	103,107,421	11.5540	Up
ENSRNOG00000005118	_	_	_	_	_	11.0079	Up
ENSRNOG00000003666	IGJ	Immunoglobulin joining chain	14	21.087.060	21,093,606	10.3672	Up
ENSRNOG00000026430	Trim58	Tripartite motif-containing 58	10	44,076,388	44,087,527	10.3524	Up
ENSRNOG00000031687	_	_	_	_	_	10.1799	Up
ENSRNOG00000010278	Il6	Interleukin 6	4	3,095,536	3,100,112	10.1410	Up
ENSRNOG00000020355	Twist2	Twist basic helix-loop-helix transcription factor 2	9	98,599,201	98,642,827	9.9621	Up
ENSRNOG00000030281	Klk10	Kallikrein related-peptidase 10	1	100,787,665	100,791,042	-10.4352	Down
ENSRNOG00000026522	_	_	_	_	_	-9.4750	Down
ENSRNOG00000037299	Smpd5	Sphingomyelin phosphodiesterase 5	7	117,337,460	117,339,998	-9.1895	Down
ENSRNOG00000009204	Il17re	Interleukin 17 receptor	4	208,711,257	208,723,904	-8.9065	Down
ENSRNOG00000020936	Nradd	Neurotrophin receptor associated death domain	8	118,231,775	118,234,748	-3.2271	Down
ENSRNOG00000042717	RGD1566380	Uncharacterized protein LOC365871	2	217,478,839	217,482,049	-2.3812	Down
ENSRNOG00000006104	Tg	Thyroglobulin	7	107,399,165	107,585,123	-2.2522	Down
ENSRNOG00000011861	Aadat	Aminoadipate aminotransferase	16	32,665,787	32,702,406	-1.9385	Down
ENSRNOG00000033844	Bex1	Brain expressed,Xlinked 1	1	117,724,202	117,725,601	-1.8810	Down
ENSRNOG00000012223	_	_	9	59,774,372	60,016,299	-1.7161	Down

### GO and pathway analysis of differentially expressed genes

In order to better understand the biological functions of the differentially expressed genes, we performed a GO and pathway analysis by using our method (details see Materials and Methods). The results of GO and pathway were ranked by p-values in ascending order (The whole list of significant functional categories can be found in S2 Table). We found that the top GO terms associated with these genes were immune system process, extracellular region part, extracellular region and biological regulation (Table 5). The top pathways associated with these genes were malaria, African trypanosomiasis and chagas disease (Table 6). These pathways were all associated with various diseases which indicated that ischemia myocardial might be related with many other diseases.

**Table 5:** Top ten of Enriched Gene Ontology(GO) terms associated with the differentially expressed genes.

GO term id	GO term name	P value
GO:0002376	Immune system process(BP)	6.59E-21
GO:0044421	Extracellular region part(CC)	2.52E-19
GO:0005576	Extracellular region(CC)	1.98E-18
GO:0065007	Biological regulation(BP)	7.72E-16
GO:0050789	Regulation of biological process(BP)	2.83E-14
GO:0031012	Extracellualr matrix(CC)	1.13E-13
GO:0023033	Signaling pathway(BP)	1.22E-13
GO:0050794	Regulation of cellular process(BP)	7.13E-13
GO:0051239	Regulation of multicellular organismal process	1.17E-12
GO:0050896	Response to stimulus(BP)	1.47E-12

**Table 6:** Top ten of pathways associated with the differentially expressed genes.

Pathway	P value
Malaria	1.11E-13
African trypanosomiasis	8.27E-11
Chagas disease(American trypanosomiasis	2.79E-10
Rheumatoid arthritis	7.19E-10
Osteoclast differentiation	1.33E-09
Cytokine-cytokine receptor interaction	2.99E-08
Leishmaniasis	4.74E-08
Cell adhesion molecules(CAMs)	1.60E-07
ECM-receptor interaction	2.66E-06
Complement and coagulation cascades	4.34E-06

### Gene structure refinement and novel transcripts prediction

For some species which were not fully studied, in order to further complete the gene annotation, we can refine gene structures with reads. Through comparing the assembled transcripts and the gene annotation, we refined structures of 9,587 genes in controls and 10,301 genes in cases.

At the same time, we compared our assembled transcripts and annotated genomic transcripts from reference sequences to discover novel transcripts. Finally, we found 1,703 and 1,552 novel transcripts in cases and controls respectively (see S3 and S4 Tables).

## Discussion

In our study, we performed a comprehensive transcriptome analysis of RNAs from normal and myocardial ischemia rats using the Illumina RNA-seq technology. The results of this study revealed the differentially expressed genes between normal and myocardial ischemia samples. Meanwhile, we also found some novel transcripts and extended the structure of many genes by comparing assembled transcripts and annotated transcripts from reference sequences.

We used the Illumina HiSeq 2000 to perform paired end run for all of our mixed samples. Paired end technology can reveal the linkage between two ends of DNA fragments[24], which helped us get more precise alignment of reads. Then we mapped all of the reads to reference genes and genome using SOAP2 and founde that the percentage of reads mapped to the reference genome were 76.33% and 77.53% in cases and controls, respectively. By comparing the FPKM of genes between normal and disease samples, we found 728 differentially expressed genes between them. Among these genes, 707 genes were up-regulated and 21 genes were down-regulated. The GO analysis indicated that these differentially expressed genes were mainly associated with immune system process, signaling pathway and response to stimulus and stress. Innate immune and inflammatory responses after transient ischemia is the most common cause of myocardial inflammation and may contribute to injury[25]. Yan et al. performed a study and demonstrated that myocardial ischemia activated an innate immune signaling through TLR4 and HSP60, which plays an important role in mediating apoptosis and inflammation during myocardial ischemia[26]. It has been reported that some signaling pathways played a key role in the myocardial ischemia[27]. For instance, the Toll-like receptor 4 (TLR4)/NF-κB signaling pathway mediates early inflammatory responses, leading to reduced inflammatory reaction and subsequently myocardial cell apoptosis[28]. At the same time, the results of pathway analysis suggested that these genes were most enriched in some diseases associated pathways. In addition, by comparing our assembled transcripts with annotated transcripts from reference genome, we found 1,703 novel transcripts in cases and 1,552 in controls. Using the same method, we refined the structures of 10,301 genes in cases and 9,587 genes in controls.

Among the top-regulated and down-regulated genes, some genes described in previous studies were associated with myocardial ischemia and they were significant up-regulated, including Cxcl2 [29] and Il6 [30, 31]. However, there were no studies directly proved that the down-regulated genes were associated with myocardial ischemia.

These findings provided a way to further study the pathogenesis of myocardial ischemia. However, our research does have limitations. Since the results of our study were mainly from bioinformatics analysis, they were needed to be confirmed by experiments. In addition, in the process of RNA-seq, bias may exist and the cDNA library may not include all expressed RNAs. Although the pathogenesis of myocardial ischemia was still unclear, our analyses indicated the changes of the genes and/or transcripts from normal to disease status. We believe that with the development of sequencing technique and bioinformatics, the findings of our study will be more accurate and our knowledge of the pathogenesis of myocardial ischemia will be expanded.

## Conclusions

Our study provides important insights into the transcriptional regulation of gene expression in rat with myocardial ischemia using RNA-seq. We found the differentially expressed genes between normal and disease samples and the functional GO terms and pathways associated with them. Additionally, we refined the structures of some genes and have predicted some transcripts. Further analysis using large size of samples will be needed to validate the differentially expressed genes in this study and illuminate the pathogenesis of myocardial ischemia.

## Supporting Information

S1 Fig Quality distribution of base along reads for cases. (PNG)

S2 Fig Quality distribution of base along reads for controls. (PNG)

S1 File NC3Rs ARRIVE Guidelines Checklist 2014. (DOCX)

S1 Table Detailed information of 707 up-regulated genes and 21 down-regulated genes. (XLS)

S2 Table Significant GO terms to which differentially expressed genes are annotated. (XLS)

S3 Table Novel transcripts in case samples. (XLS)

S4 Table Novel transcripts in control samples. (XLS)
