# Supplementary figures and images for: Transcriptomic Analysis of Myocardial Ischemia Using the Blood of Rat
Source: PLoS One. 2015 Nov 5;10(11):e0141915. doi: 10.1371/journal.pone.0141915 (PMC4634849; doi:10.1371/journal.pone.0141915)

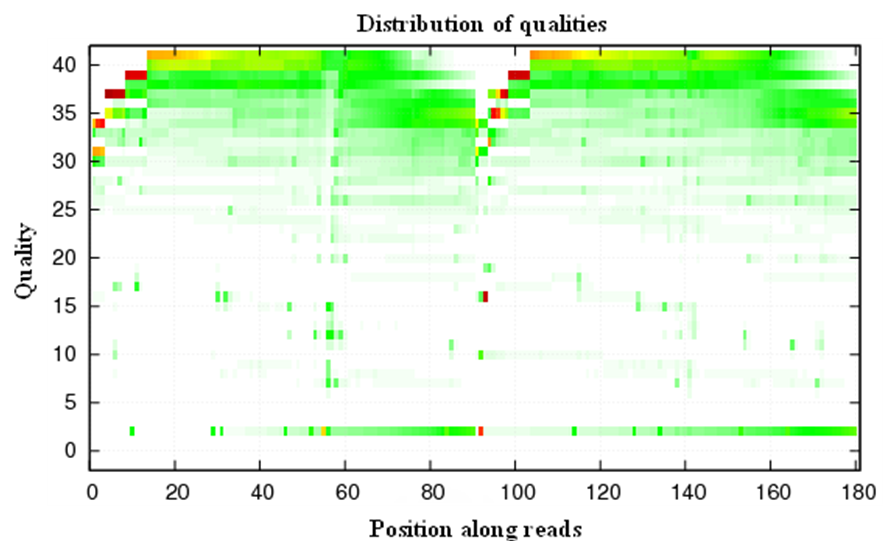

Supplement: S1 Fig — (PNG) [file pone.0141915.s001.png]

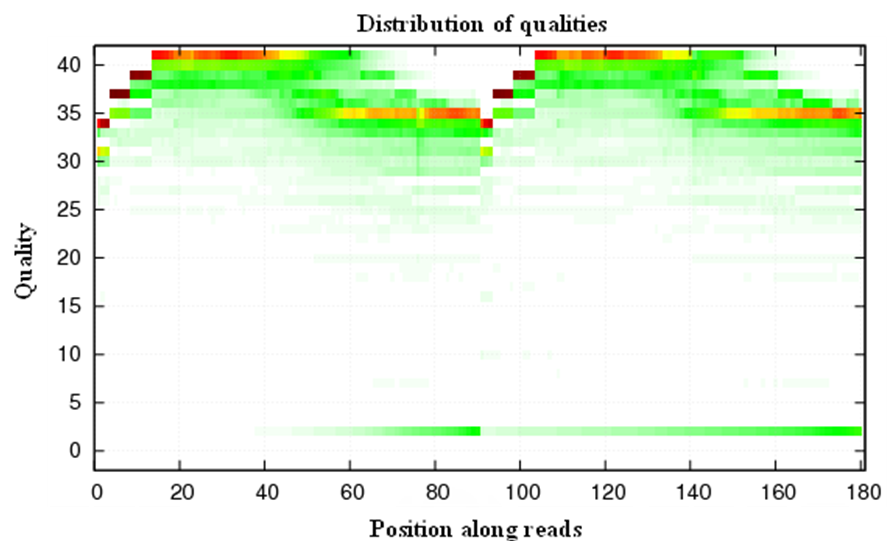

Supplement: S2 Fig — (PNG) [file pone.0141915.s002.png]
